# Knowledge about diabetes in Malmö prior to initiation of “Cities Changing Diabetes”

**DOI:** 10.3389/fpubh.2024.1522549

**Published:** 2025-01-16

**Authors:** Magdalena Annersten Gershater, Margareta Rämgård, Cecilia Nagorny Holmberg, Mathias Grahn, Slobodan Zdravkovic

**Affiliations:** ^1^Faculty of Health and Society, Department of Care Science, Malmö University, Malmö, Sweden; ^2^Novo Nordisk Scandinavia AB, Malmö, Sweden; ^3^City of Malmö, Stadskontoret, Unit for Statistics and Analysis, Malmö, Sweden

**Keywords:** diabetes mellitus, home care, migration, public health, school health care

## Abstract

**Aim:**

To identify existing public knowledge regarding diabetes and diabetes-related services offered to persons living with diabetes in the City of Malmö.

**Methods:**

A literature review of City of Malmö’s website, public statistics, School health documentation, job databases, education programs, local newspaper, Swedish National Diabetes Register, and *PubMed* was performed in 2020.

**Results:**

We identified political decisions about diabetes nurses in home care, financing a project about diabetes complications, and funding support in schools for designated children. Schools had no registrations of diagnoses. Diabetes was common among pregnant women. The local newspaper discussed children and older people with diabetes asking for increased support. Job listings did not require diabetes-relevant competencies. Curricula for nursing assistants did not mention diabetes. National Diabetes Register reported 16,658 persons in Malmö. Three articles were identified in *PubMed*.

**Conclusion:**

Public documents in Malmö did not mention diabetes despite being responsible for caring for persons with diabetes.

## Background

In Sweden, the prevalence of diabetes is estimated at 5% (1) and its incidence at 4.4 per 1,000 (2). In addition, an unknown number of persons live with undetected type 2 diabetes (3). How this is distributed over the country is not known. The development of the population in Sweden’s third largest city, Malmö, has for many years been driven by the preference of immigrants residing in Sweden to live in this city, making it one of the country’s most multicultural cities (4). The prevalence of diabetes in the population of Malmö is not exactly known, but several of the inhabitants born in Middle Eastern countries, where diabetes is more common, may have moved in with already diagnosed diabetes (5). The prevalence of type 2 diabetes is driven by genetic factors (6, 7) and a societal development toward a sedentary lifestyle in combination with more caloric value dietary habits. At the time of this study, there was no systematic screening to detect type 2 diabetes in the region, but healthcare centers in Malmö perform opportunistic screening when patients seek medical care for other health-related issues. Diabetes care in Sweden is organized as follows: All children with diabetes are managed by pediatric physicians and specialist nurses working in pediatric clinics, and hospitals are responsible for adult persons with type 1 diabetes and persons with complicated diabetes type 2. Meanwhile, persons with type 2 diabetes are mainly managed by general practitioners at the primary healthcare centers. Health and Social Care Department in Malmö provide care for people who are unable to maintain their own self-care, including older adult care, social-psychiatric care and care for persons with intellectual disabilities, and nursing interventions for persons who have been treated at the hospital but cannot take care of their health-related needs after discharge (8). City of Malmö is responsible for medical care by healthcare professionals up to the level of registered nurses. General practitioners work in the Region’s healthcare centers and are available as consultants (9). Arguably, several of those persons in need for daily complex nursing interventions have diabetes and are living with multiple diabetes complications.

The Cities Changing Diabetes project was launched in 2014 by Novo Nordisk together with several cities around the world as a systematic method to reduce the increasing incidence of diabetes and its complications (10). City of Malmö has decided to become a partner of this project, with its overarching theme to find a way to stem the tide of an evolving diabetes epidemic. According to the model of Cities Changing Diabetes, a mapping of the diabetes situation in each city should be performed prior to implementation of any interventions. Therefore, the aim of the present study was to identify existing public knowledge regarding diabetes and diabetes-related services offered to persons living with diabetes in the City of Malmö.

## Method

This is a general literature review of published public information regarding diabetes in City of Malmö. The following sources were assessed in October 2020 using the search term “diabetes”: The search engines of City of Malmö’s website and archive were used to identify any diabetes relevant information and public statistics. Administration of Compulsory Education Department was approached to ask for diabetes prevalence among school children in Malmö. To identify what healthcare services Health and Social Care Department and Disability Support Department offer to persons with diabetes, we performed a search for “diabetes” on the city’s website and its subpages regarding home nursing and assisted-living facilities. Malmö City’s office was also contacted via telephone.

City of Malmö’s job database was searched for any position that had “diabetes” in its qualifications. In addition, the catalog for upper secondary school nursing assistant program and adult education programs were searched for diabetes related content. The search engine of the major local newspaper Sydsvenskan was also used to retrieve any diabetes related articles about Malmö.

Data was extracted from healthcare centers located in Malmö using the Swedish National Diabetes Register reports. Data from the Skåne University Hospital in Malmö recorded in the NDR were not possible to use as the Malmö population could not be extracted from the hospital’s catchment area, which also covers several other municipalities.

Finally, a search was performed in *PubMed* using the search terms “diabetes” and “Malmö.”

## Ethics

There is always a risk for the invasion of privacy when registers are analyzed. However, in this literature review, all results are based on the gathering of already published data. Thus, no individuals are identified except for those mentioned in the newspaper articles, but their names are not mentioned in this review.

## Results

### City of Malmö website

At City of Malmö official website, we found no diabetes-related information available, other than a press-release about the present Cities Changing Diabetes project.

### City of Malmö’s archive: public statistics and reports

In the archive, the following publications were identified: A decision by the City Executive Committee from 2006 presents the decision to strengthen the care chain in diabetes, where City of Malmö would appoint one diabetes-competent nurse to act as a link between care givers and arrange local diabetes-related educational activities for registered nurses in collaboration with the region’s primary care (KS-SDF-2006-00063). In 2012, a questionnaire about rehabilitation for patients with diabetes and stroke was presented to the decision makers. There were also several applications and decisions regarding increased funding for extra support in school for designated children with diabetes.

Another document found in the archive is the Commission for a Socially Sustainable Malmö report (4), which focused on social determinates rather than medical diagnoses. According to the report, studies on pregnant women in Malmö indicated that obesity and diabetes are risk factors for birth complications and that these factors were predominantly seen among the poorer population in the city. The report also mentioned that diabetes and cesarean section rates were pronounced risk factors for maternal morbidity. Therefore, great efforts should be devoted to identifying mothers with diabetes during pregnancy through basic maternal care (11). Other cited studies performed in Malmö investigated the association between obesity with maternal and fetal complications and revealed that maternal obesity is strongly related to the development of diabetes, venous thromboembolism, and cardiovascular disease in pregnancy (12, 13), as well as accelerated fetal growth; the latter causes both serious complications for the future child, including pre-delivery fetal death (14), and severe birth defects in the mother (15). Obesity contributes not only to an increased cesarean section rate but also to increased complication risks, such as bleeding and infections (16, 17). There were statistically significant differences between Swedish-born women in Malmö, on the one hand, and women coming from Iraq and Lebanon, on the other: 2% of Swedish-born women and 9% of women from Lebanon and Iraq who were pregnant with their second child in Malmö had type 2 diabetes. Rosengård, Oxie, Kirseberg, and Fosie were over-represented districts for obese mothers (BMI > 30) Oxie being significantly over-represented in both underweight and overweight children (11).

### Administration of compulsory education department

City of Malmö school health records of all children aged 6–19 years, which are used by the school nurses for documentation, did not systematically record any medical diagnoses of the children.

### Health and social care department

Registered nurses working in the Health and Social Care Department use the electronic record system Pro Capita for documentation in home care or assisted living facilities. It was not possible to extract any medical diagnoses on a group level from Pro Capita.

### City of Malmö’s job database

A search in Malmö’s job database on January 24, 2020, revealed five open positions for healthcare assistants, eight for registered nurses, two for physiotherapists, and one each for occupational therapist and psychologist. None of the job advertisements mentioned the word “diabetes” or any diabetes-related competency under “Qualifications.”

### Curricula for healthcare assistants and healthcare adult education programs

Three programs were identified. In none of the presentations of the programs or in the curricula was the word “diabetes” mentioned, nor was there mention of any other chronic condition connected to diabetes.

### The local newspaper Sydsvenskan

A search in Sydsvenskan’s search engine for the word “diabetes” and the year 2020 resulted in 18 hits containing general diabetes information or general articles regarding diabetes studies performed elsewhere in the world, mainly regarding type 1 diabetes. The same search for the year 2019 yielded 77 hits including one article about an older adult woman in Malmö with diabetes and chronic obstructive pulmonary disease who did not receive sufficient social support (18). The remaining diabetes-related text were general articles about diabetes without any connection to Malmö. The search for 2018 yielded 86 hits among these two articles about young persons living in Malmö with type 1 diabetes (19, 20) and one about an old man who was denied moving into assisted living in Malmö because he lived with diabetes complications, but the social home service was perceived insufficient (21). The remaining diabetes-related articles concerned diabetes studies performed elsewhere in the world or general diabetes information.

### The Swedish national diabetes register

Data from the National Diabetes Register from the year 2018 showed that 16,658 persons (9,225 males and 7,433 females) had been diagnosed with diabetes at a healthcare center in Malmö.

### PubMed

Three articles were identified in the database about a population residing in Malmö. They were based on the Swedish Malmö Diet and Cancer Study cohort (26,615 adults, aged 45–74 years, 60% women). and the articles reported that a curvilinear association was observed between leisure-time, physical activity, and the risk of type 2 diabetes (22), that a lifestyle-related lipidomic profile strongly predicts the development of type 2 diabetes beyond current risk factors (23), and that a dietary pattern with health-conscious food choices was associated with a lower risk of cardiometabolic diseases in both genders (24).

## Discussion

This review to identify existing knowledge regarding diabetes in Malmö and diabetes-related services offered to persons with diabetes living in Malmö revealed that very little is known in this particular context. In public documents available on the internet, there was not much information to retrieve regarding the diabetes situation in Malmö. There were no data available in public documentation from City of Malmö regarding the prevalence of diabetes or public health activities to prevent or treat diabetes. There were some indications in the local newspaper and in the archive documents that persons with diabetes-related complications are present among care recipients in the Health and Social Care department and that there was a need for funding to support school children with type 1 diabetes. However, it was not possible to retrieve data on how many these persons were or how many children living with diabetes go to school in Malmö. This lack of information hampers the possibilities to offer good and safe care for the people residing in Malmö (25) and a safe school environment (26). Children who need special support in their development must be provided with this support. Allocating appropriate resources is difficult without knowing how many children have diabetes and need special support (26). Without this information, no systematic follow up on quality can be provided. The fact that social and healthcare services and school health services do not have a system to identify how many persons with diabetes are under their care is worrying. There is no chance to ensure proper and safe care according to the health legislation under which these services operate (25). This situation can partly be explained by the fact that social and health care work hand in hand under separate legislations: The administration is managed by social workers without medical knowledge about the particular medical needs of persons with diabetes, whether those attending school or receiving home nursing services. As the number of persons with diabetes is not known, City of Malmö has not made demands for specific diabetes competency when advertising for registered nurses, school nurses, or healthcare assistants. Nor is diabetes mentioned in City of Malmö’s own high school curricula as a requirement for healthcare assistants. How the City intends to keep a good quality of care for school children with diabetes and for persons receiving home care without knowledge of diabetes prevalence or competent staff is a mystery.

In the Commission for a Socially Sustainable Malmö report by Stigendahl et al. (4), diabetes was described as a risk factor for pregnant women and their babies. As the population in Malmö is relatively young, has many women in child-bearing age, and has high birth rates, this should be taken seriously by City of Malmö. Healthy mothers are more likely to work, whereas mothers with health issues may require extra support from social and healthcare services and their children might be in need for extra resources in school. The report also presented data on which country of origin involved higher risk and which neighborhoods had higher rates of obese mothers (4). We were not able to find any public documentation regarding whether or how City of Malmö has responded to the findings of this report.

There are several high schools that offer education for healthcare assistants in Malmö. The fact that diabetes has been invisible in the curricula and that diabetes-related competency has not been required in advertised positions for healthcare assistants is concerning. Persons with diabetes and multiple complications need care from professionals with adequate competency (27). However, the results of this review indicate that persons with diabetes who are dependent on others for maintaining their selfcare are receiving social or health care from persons with very little diabetes-related competency. This lack of specific knowledge about diabetes has also been shown in previous studies, where the documentation of diabetes-related activities in municipal home care for persons with diabetes and complications was found unsatisfactory (28, 29).

In the local newspaper, there were indications that the needs of older people and children with diabetes have not been fully met by City of Malmö. During 2018–2020, only one article was about living with type 2 diabetes, while the remaining diabetes-related news focused on persons with type 1 diabetes. This reveals that type 2 diabetes can be considered an invisible condition in the eyes of the public. Altogether, 90% of all persons living with diabetes have type 2 (1). The NDR contains records of 16,658 persons with type 2 diabetes at healthcare centers located in the city in 2018. This information should have been made public.

The relevant scientific articles showed that life-style factors affect diabetes incidence in persons living in Malmö. However, these articles involved a small sample that did not include the foreign-born population that moved into the city after inclusion in the Malmö Diet and Cancer Study cohort had closed (30).

In the first phase of the Cities Changing Diabetes project, the city-specific diabetes burden and its sociocultural drivers were mapped, either using existing public health data or through a household survey (10). Regarding other cities in the project, results from the nearby city of Copenhagen showed that there were demographic and socioeconomic differences in the prevalence of risk factors, occurrence, and treatment of diabetes. For instance, high risk was especially prevalent among those with low education and no employment. Moreover, obesity and physical inactivity were more prevalent among people with a non-Western background (31). This review study was initiated in Malmö as part of the first phase, but by using existing public health data from Malmö, the study has not been able to fully map the city-specific burden and socio-cultural burdens.

In conclusion, City of Malmö has until now lacked knowledge of diabetes and its implications for its residents, nor has it taken any action to meet their needs of prevention or provide good quality of care (mainly for persons with severe diabetes-related complications and at the end of life), for which the City is responsible.

Implications for Policy & Practice.

- City of Malmö needs to explore the burden of diabetes.
- City of Malmö home care organization is responsible for many people with diabetes and complications.
- City of Malmö needs to educate their health care staff working in school health organizations and home care about prevention of diabetes and living with diabetes.

## Links used for searches

Search engines: (www.malmo.se) and (https://malmo.se/stadsarkivet).

City of Malmö’s job database (https://malmo.se/Jobb.html).

Press release: https://malmo.se/Huvudnyheter/2019-11-15/−Malmo-ansluter-sig-till-globalt-diabetesprogram.html.

Swedish National Diabetes Register (NDR) reports (www.ndr.se/knappen).

Sydsvenska Dagbladet AB (www.sydsvenskan.se).

## Articles from Sydsvenskan

Glimberg M & El-Alawi H. Han är för pigg för att bo på äldreboende Sydsvenskan 27 April 2018 pages 2–3.

Persson A. Pensionär hemlös efter brand – får ingen hjälp. Sydsvenskan 30 September 2019 p B3.

Skeppstedt L & Brundin L Han bad om hjälp – ingen ringde 112. Sydsvenskan 2018-12-16 page B8.

Sundberg U & Frennesson S Familjen lever med diabetes dygnet runt Sydsvenskan 2018-11-14 page 30.

## Political decisions from Malmö city executive committee

KS SDF 2006 00063 Förstärkt vårdkedja vid diabetes.

## Data Availability

The raw data supporting the conclusions of this article will be made available by the authors, without undue reservation.
